# Investigation of symptom-specific functional connectivity patterns in Parkinson’s disease

**DOI:** 10.1007/s10072-025-08287-4

**Published:** 2025-06-14

**Authors:** Ani Kicik, Ali Bayram, Emel Erdogdu, Elif Kurt, Dilek Betul Saridede, Sevim Cengiz, Basar Bilgic, Hasmet A. Hanagasi, Esin Ozturk-Isik, Hakan Gurvit, Erdem Tuzun, Tamer Demiralp

**Affiliations:** 1 Department of Physiology, Faculty of Medicine, Demiroglu Bilim University, Istanbul, 34394 Turkey; 2 Hulusi Behcet Life Sciences Research Laboratory, Neuroimaging Unit, Istanbul University, Istanbul, 34093 Turkey; 3 Department of Neuroscience, Aziz Sancar Institute of Experimental Medicine, Istanbul University, Istanbul, 34093 Turkey; 4 Department of Psychology, Faculty of Economics and Administrative Sciences, Isik University, Istanbul, 34980 Turkey; 5 Department of Biomedical Engineering, Faculty of Engineering and Natural Sciences, Istanbul Atlas University, Istanbul, 34403 Turkey; 6 College of Technological Innovation, Zayed University, Dubai, 144534 UAE; 7 Behavioral Neurology and Movement Disorders Unit, Department of Neurology, Istanbul Faculty of Medicine, Istanbul University, Istanbul, 34093 Turkey; 8 Institute of Biomedical Engineering, Bogazici University, Istanbul, 34684 Turkey; 9 Department of Physiology, Istanbul Faculty of Medicine, Istanbul University, Istanbul, 34093 Turkey

**Keywords:** Parkinson’s disease, Magnetic resonance imaging, Functional connectivity, Visuospatial functions, Cognitive impairment

## Abstract

Parkinson’s disease (PD) is a complex neurodegenerative disease, characterized by pronounced heterogeneity in symptoms. This study investigates the functional connectivity (FC) patterns associated with distinct symptom clusters, aiming to elucidate the heterogeneity in PD and uncover the neural mechanisms underlying its motor and cognitive symptoms. Resting-state functional MRI (rs-fMRI) data from 55 non-demented PD patients and 24 healthy controls (HC) were used to perform seed-to-seed FC analyses. A clustering algorithm was applied to the cognitive and motor scores of all PD patients to generate relatively homogeneous symptomatic subgroups. PD patients exhibited a general decrease in FC within a network comprising the sensorimotor network (SMN) and the visual network (VN) regions. Symptom-based clustering revealed three relatively homogeneous subgroups, exhibiting a gradient pattern: patients with greater motor deficits showed significant disconnection within the SMN, whereas patients with greater visuospatial deficits exhibited reduced FC in an extended subnetwork, with pronounced disconnections between the VN and SMN areas. Our study demonstrated a notable disconnection between the SMN and VN, indicating impaired visual-motor integration in PD. Stronger disconnection within the SMN was associated with greater motor dysfunction, and stronger visual-sensorimotor disconnections were associated with greater visuospatial deficits. These findings suggest that at least two separate routes of functional disconnection may be responsible for the inhomogeneous symptom distribution in PD.

**Supplementary Information:**

The online version contains supplementary material available at 10.1007/s10072-025-08287-4.

## Introduction

Parkinson’s disease (PD) is one of the most common neurodegenerative diseases characterized by motor findings including resting tremor, bradykinesia, rigidity and postural instability [1]. Degeneration of dopaminergic neurons in the substantia nigra pars compacta and the resulting dysfunction in the cortico-striatal-thalamic-cortical loop are the pathological hallmarks of PD, which cause motor symptoms of the disease [2]. Although the diagnostic criteria for PD include motor findings, a wide variety of non-motor symptoms may also occur in PD patients.

Cognitive impairment is one of the essential non-motor symptoms of PD, leading to functional impairment and having a significant negative impact on patients’ quality of life [3, 4]. The spectrum of cognitive impairment in PD ranges can be observed as extending from normal cognition through subjective cognitive impairment (PD-SCI) and mild cognitive impairment (PD-MCI) to PD dementia (PD-D). Several long-term studies have indicated that patients with PD-MCI have an increased risk of developing PD-D [5]. Pedersen et al. reported in a five-year follow-up study that 60% of patients with PD-MCI progressed to PD-D [6].

Since cognitive impairments have clinical relevance in the evolution of PD-D, a large number of FC studies have focused on investigating the impact of brain networks on cognitive impairments. A significant number of researchers have explored intranetwork and internetwork connectivity changes, with particular attention to the default mode network (DMN), dorsal attention network (DAN), fronto-parietal network (FPN), sensorimotor network (SMN), and visual network (VN), which are associated with cognitive impairment in PD. These studies found FC alterations within and between different brain networks and identified relationships between FC and clinical and cognitive scores in PD [7–11]. Likewise, some FC studies have concentrated on subcortical structures, particularly the striatum, to examine its role in motor impairments in PD [12–14]. While the literature contains numerous neuroimaging studies linked to both cognitive and motor impairments in PD, the findings remain notably heterogeneous. The varying findings across studies emphasize the heterogeneity in both the cognitive profile and the pathophysiology of PD, which remains incompletely clarified [15, 16]. Heterogeneous cognitive profiles in PD, as well as differences in the criteria used to diagnose PD-MCI and methodological differences in neuroimaging studies, appear to be the main reasons for the discrepancies between reports [17]. Understanding the heterogeneity in PD and uncovering the neural mechanisms underlying motor and cognitive symptoms is important for enabling the development of targeted treatment approaches and potentially disease-modifying interventions [18].

In this study, we aimed to identify functional changes in the brain associated with motor symptoms and two distinct cognitive symptoms, trying to unravel the complexity arising from heterogeneity in PD. Research on PD has identified two distinct profiles of cognitive decline, as highlighted in the dual syndrome hypothesis. The first profile involves frontostriatal dysfunctions driven by dopamine depletion, leading to impairments in executive functions such as attention and planning. The second profile is characterized by posterior cortical deficits, including difficulties in visuospatial processing, memory, and language, which are more strongly linked to an increased risk of progression to dementia due to disruptions in cholinergic pathways [16–19]. Based on this hypothesis, we addressed executive and visuospatial cognitive impairments in PD, which may indicate two distinct underlying neuropathologies and evaluated patients in terms of these two cognitive subtypes in PD. In addition to examining the functional effects of these two cognitive symptoms, we incorporated the impact of motor impairment severity in PD into FC analyses. To overcome the complexity arising from the cognitive heterogeneity in PD, we planned to use two different approaches. We first explored the possible relationships between the cognitive and motor scores and the FC metrics, and secondly, used a clustering approach aiming to generate homogeneous subgroups in terms of the severity of these three symptom types in PD. Through these approaches, we aimed to first identify FC changes in non-demented PD patients compared to healthy controls (HC). Furthermore, we sought to determine more specific FC patterns associated with executive and visuospatial cognitive and motor symptoms, using homogeneous subgroups generated through the clustering approach. We hypothesized that functional changes in PD would be specifically associated with the severity of cognitive and motor symptoms, with FC alterations becoming more pronounced as symptom severity increased.

## Materials and methods

### Participants

Fifty-five non-demented patients with idiopathic PD and 24 HC, all between 45 and 85 years of age, were included in this study. All PD patients were recruited from Behavioral Neurology and Movement Disorders Unit of the Department of Neurology at the Istanbul Faculty of Medicine, Istanbul University, Turkey. The diagnosis of PD was made according to the UK Parkinson’s Disease Society Brain Bank Criteria [20]. The non-demented PD group consisted of patients who did not meet the diagnostic criteria for PD-D as defined by Emre et al. (2007) [21], and who had a Clinical Dementia Rating (CDR) global score ≤ 0.5 and a CDR-Sum of Boxes (CDR-SOB) score ≤ 2.5. Neuroimaging, neuropsychological and motor assessments were performed while PD patients were taking their usual antiparkinsonian medication (ON state) which was calculated as the Levodopa equivalent daily dose (LEDD) [22]. The exclusion criteria were as follows: (1) dementia at diagnosis [21] (2) Mini-Mental State Examination (MMSE) score < 25; (3) History of stroke or head injury; (4) Significant structural brain abnormalities other than mild white-matter hyperintensities observed in FLAIR images; (5) Neurological comorbidity; (6) Hoehn and Yahr (HY) score > III; and (7) Significant psychiatric or systemic disease (including depression as defined by a Geriatric Depression Scale (GDS) score of > 14), or an unstable medical condition.

### Clinical and neuropsychological assessments

Motor disease severity was assessed using the Unified Parkinson’s Disease Rating Scale Part III (UPDRS-III). All participants completed the MMSE to measure global cognition. In addition, PD patients underwent the Stroop and Benton Judgment of Line Orientation (JLO) tests to evaluate executive and visuospatial functions, respectively.

### MRI acquisition

MR images were acquired on a 3T Philips MRI scanner (Achieva, Philips, The Netherlands), equipped with a 32-channel SENSE head coil at the Istanbul University Hulusi Behçet Life Sciences Research Laboratory Neuroimaging Unit. Functional images were obtained using a T2*-weighted echo planar imaging sequence with the following parameters: repetition time (TR) = 2000 ms, echo time (TE) = 30 ms, field of view (FOV) = 224 × 240 mm, number of slices = 36, slice thickness = 1 mm (without gap), voxel size = 2 × 2 × 4 mm, flip angle = 77°. During the functional MRI scan, participants were instructed to stay awake with their eyes closed. High-resolution structural images were acquired using a T1-weighted turbo field echo sequence (TR = 8.4 ms, TE = 3.9 ms, 180 axial slices, FOV = 250 × 250 mm, 1 mm isotropic voxel, flip angle = 8°). T2-weighted FLAIR sequence (TR = 4800 ms, TE = 259 ms, TI = 1650 ms, FOV = 250 × 250 mm, number of slices = 60, voxel size = 1.11 × 1.11 × 3 mm^3^) was also obtained to evaluate white matter hyperintensities.

### Preprocessing of functional data

Preprocessing analysis was performed using SPM12 (Statistical Parametric Mapping version 12; http://www.fil.ion.ucl.ac.uk/spm/) and the CONN functional connectivity toolbox version 18.b (https://www.nitrc.org/projects/conn) [23]. Initially, the functional volumes were realigned to the first volume to correct for head movement. Outlier volumes were then identified based on the framewise displacement (FD) and global signal (GS) values using the Artifact Detection Tools (ART), implemented in CONN toolbox [24]. The GS threshold was set at 5 standard deviations, and the FD threshold was set at ≥ 0.9 mm, representing the difference in composite motion of an image from the previous scan. Five patients out of 60 were excluded from further analyses due to the number of outlier volumes exceeding 20% (> 43) of the total number of functional volumes (214). In order to test the significance of head movement differences between PD patients and the HC group, the mean FD, the number of outlier scans, the mean motion, and the mean GS were calculated for each participant and compared between groups using the Mann-Whitney U test. In the next step, co-registration of functional data to structural images and segmentation procedures were performed. Structural images were segmented into white matter (WM), gray matter, and cerebrospinal fluid (CSF) tissue classes and then normalized to the Montreal Neurological Institute (MNI) standard space. The functional data were spatially normalized using the resulting transformation matrix and resampled to a final isotropic voxel size of 2 mm^3^. Then, functional data were smoothed using a Gaussian kernel of 8 mm full width at half maximum. The denoising procedure was applied to functional data in order to remove noise factors due to motion and physiological effects, such as respiration and pulsation. For denoising, signals from the white matter and CSF, motion parameters, and ART-based scrubbing parameters, and the effect of the scanning session were regressed out. Finally, functional data were processed with a band-pass filter of 0.01–0.1 Hz, and linear detrending was applied.

### Functional connectivity analysis

Seed-to-seed functional connectivity analysis was performed using the CONN toolbox. The Automated Anatomical Labeling 3 (AAL3) atlas was used to define the regions of interest (ROIs). A total of 112 ROIs were used from the AAL3 atlas, excluding the cerebellum and the subdivisions of the thalamus (the entire thalamus region was included from the AAL2 atlas). For the connectivity analyses, initially, the BOLD time series were extracted by averaging the time series within each ROI. Bivariate ROI-to-ROI connectivity matrices were created for each subject, and then Pearson’s correlation coefficients were converted to Z-scores using Fisher’s Z transformation for further analyses. The Network-Based Statistics (NBS) method, known for its high statistical power, was employed for all FC analyses in PD to examine changes in FC [25]. In the first step, PD and HC groups were compared by applying a two-sample t-test for each ROI-to-ROI connection separately. A p-value of < 0.001 (uncorrected) was applied as a primary threshold constructing a set of suprathreshold connections. For each suprathreshold subnetwork, a family-wise error (FWE)-corrected p-value was calculated using a permutation test (10,000 iterations). FWE-corrected p-values of < 0.05 (at the network level) were accepted as significant. Resulting suprathreshold subnetworks were determined for statistical significance based on their connectivity.

Besides group comparisons, two different approaches were implemented to explore the effects of motor and cognitive performance in the PD group using subject-specific ROI-to-ROI functional connectivity values. In the first approach, an NBS-based linear regression analysis was conducted across the entire PD group using all 112 ROIs from the AAL3 atlas. This analysis examined the associations between functional connectivity and Stroop interference, JLO, and UPDRS-III scores, which served as regressors representing executive, visuospatial, and motor functions, respectively. In the second approach, Stroop interference, JLO and UPDRS-III scores were used for the unsupervised classification of PD patients into subgroups. Then, statistically significant connectivity differences between each of these subgroups and the HC group were tested to demonstrate the cognitive and motor aspects of connectivity changes. The construction of PD subgroups was performed by implementing the spectral clustering algorithm (SCA) included in the Scikit-learn (version 0.23.7) machine learning library for Python [26]. To prevent the domination of one score over others in the classification task, each score was standardized by removing the mean and scaling it to unit variance. Before the implementation of SCA, the optimal number of clusters was identified by examining the eigenvalues of the affinity matrix, which was constructed by computing the graph of nearest neighbors with the Euclidean distance metric. The index of the highest gap between consecutive eigenvalues (*n* = 3) was set as the optimal number of clusters and used while implementing SCA [27]. The comparison of each PD subgroup with the HC group was performed using two-sample t-tests limited to the set of ROIs found to be affected in the PD vs. HC group comparison. Statistical inference for both regression analyses and classification-based group comparisons was conducted using the NBS method, applying a primary threshold of *p* < 0.001 for edges and a family-wise error (FWE)-corrected threshold of *p* < 0.05 at the network level, consistent with the PD vs. HC group comparisons.

### Statistical analysis

Statistical analysis of demographic and clinical data was conducted using IBM SPSS 25. A two-sample t-test was employed for the analysis of normally distributed data, while the Mann-Whitney U test was applied to non-normally distributed data. To assess whether disease duration was associated with the clustering variables (UPDRS-III, Stroop, and JLO scores), Spearman correlation analysis was performed. Gender differences were assessed using Pearson’s chi-squared test. Statistical significance was accepted as *p* < 0.05.

## Results

### Demographic and clinical characteristics of participants

Seventy-nine participants were enrolled: 55 PD patients and 24 HC. PD and HC groups did not differ in age, education, and gender. Although PD patients were non-demented, MMSE scores in PD patients were significantly lower than those of the HC group (*p* < 0.001). There was no significant difference between the groups in terms of GDS scores (*p* = 0.123). The demographic and clinical characteristics are presented in Table 1.

**Table 1:** Demographic and clinical characteristics of PD patients and healthy controls

	HC Mean (SD) (*n* = 24)	PD patients Mean (SD) (*n* = 55)	HC vs. PD patients Statistics, *P* value
Gender (female/male)	9/15	16/39	*X*^*2*^ = 0.546, 0.460
Age	59.79 (7.12)	61.95 (8.62)	*t*(77) = -1.073, 0.287
Education (years)	10.04 (4.03)	9.49 (3.85)	*U* = 615, 0.621
GDS	4.33 (3.37)	5.93 (3.97) (*n* = 54)	*U* = 506, 0.123
MMSE	29.67 (0.81)	28.78 (1.25)	*U* = 351.5, < 0.001
Disease duration (years)	NA	6.18 (3.67)	
LEDD	NA	732.14 (362.8) (*n* = 54)	
UPDRS-III	NA	28.82 (12.05)	
Stroop interference score	NA	63.47 (32.90)	
JLO	NA	22.62 (5.08)	

SD: Standard Deviation, GDS: Geriatric Depression Scale, MMSE: Mini-Mental State Examination, UPDRS-III: Unified Parkinson’s Disease Rating Scale Part III, LEDD: Levodopa Equivalent Daily Dose (mg/day), JLO: Benton Judgment of Line Orientation, X^2^: Chi-Square test, t: Two-Sample t-Test, U: Mann-Whitney U Test, PD: Parkinson’s Disease, HC: Healthy Control

### Functional connectivity results

**Fig. 1:**
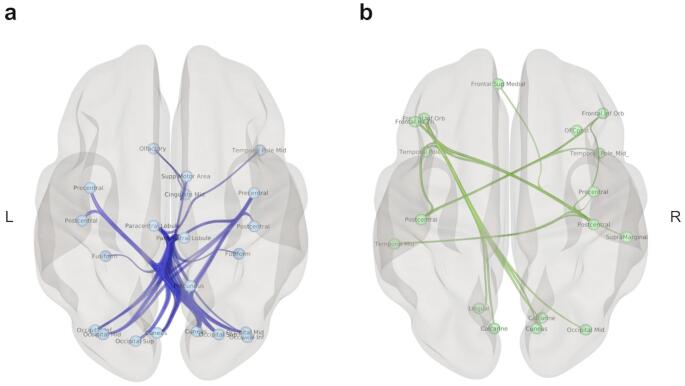
Subnetworks identified with NBS are presented. **a**) The subnetwork depicts decreased connectivity in PD patients compared to the HC group. **b**) The subnetwork depicts a negative regression with Stroop test scores in the PD group. Edges between brain regions represent functional connections. L: Left, R: Right

In the preprocessing steps, no statistically significant difference was found between PD patients and the HC group in terms of the mean FD (*U* = 620, *p* = 0.670), mean motion (U = 608, *p* = 0.579), number of outlier scans (*U* = 601, *p* = 0.499), and mean GS (*U* = 537, *p* = 0.190). Seed-to-seed analysis was performed to explore changes in FC between PD patients and the HC group using the NBS method. The network-level NBS identified a subnetwork showing significantly decreased connectivity in PD patients compared to the HC group (*p* < 0.05, FWE-corrected). This subnetwork contained 21 cortical regions defined by the AAL3 atlas, including mostly sensorimotor and visual regions, in addition to the precuneus, middle cingulate gyrus, right temporal pole, and olfactory regions. This subnetwork covered sensorimotor regions, including the precentral, postcentral, and paracentral regions, and supplementary motor area (SMA), as well as visual regions including the superior, medial, and inferior occipital regions, cuneus, and fusiform gyrus. All regions in this subnetwork are listed in Supplementary Material Tables 1 and displayed in Fig. 1a in detail.

In addition to the group comparison, a second line of NBS analyses was performed within the PD group for the regressions of FC with Stroop interference, JLO, and UPDRS-III scores. While no significant FC regressions were found with JLO and UPDRS-III scores, a subnetwork was identified, showing a negative regression with Stroop interference scores (*p* < 0.05, FWE-corrected). This subnetwork included 17 brain regions, comprising frontal and temporal regions, in addition to visual and sensorimotor areas, including the cuneus, calcarine, middle occipital, precentral, and postcentral regions (for details, please see Supplementary Material Tables 2 and Fig. 1b).

**Fig. 2:**
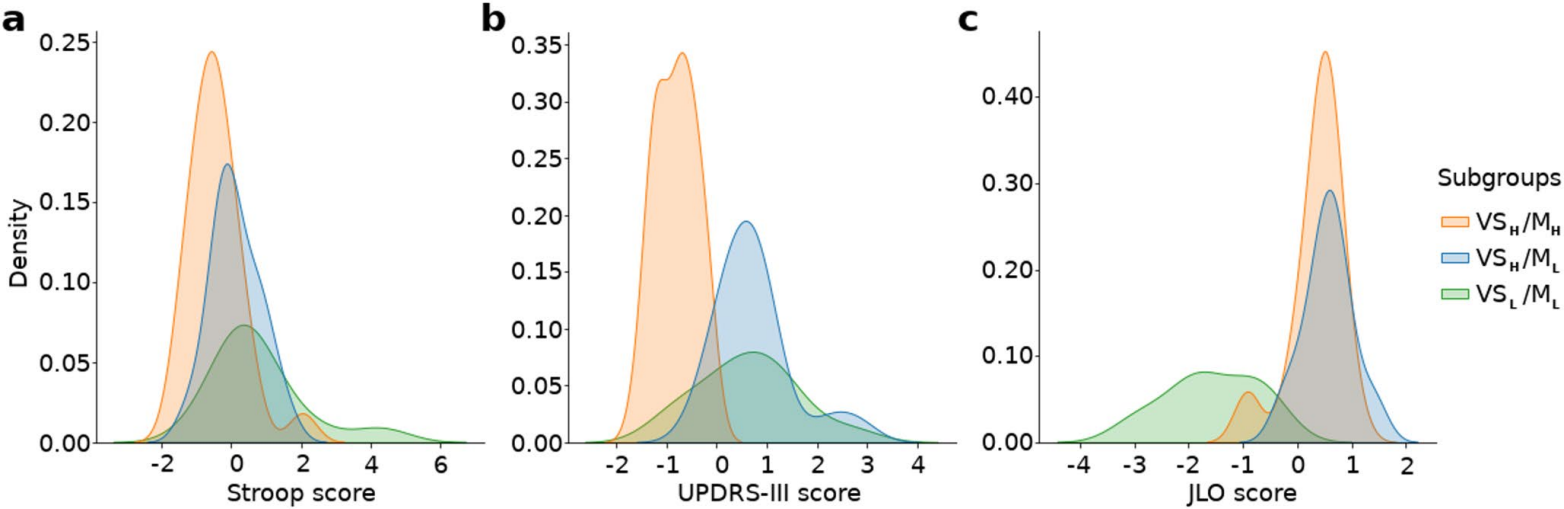
Histogram plots of the three subgroups separated by a clustering algorithm based on neuropsychological and motor test scores. **a**) Distribution of Stroop scores. **b**) Distribution of UPDRS-III scores. **c**) Distribution of JLO scores. The histogram, coded in orange, represents PD patients with low UPDRS-III scores and high JLO scores, indicating that this subgroup is characterized by higher visuospatial and motor function compared to the other two subgroups (VS_H_/M_H_). The histogram coded in blue represents PD patients with lower motor function but preserved visuospatial function (VS_H/_M_L_). Lastly, the histogram coded in green represents PD patients with lower visuospatial function compared to the other two subgroups, who also exhibit lower motor function (VS_L_/M_L_)

Considering the heterogeneous character of functional deficit patterns in PD subjects, in terms of various degrees of coexistence of functional declines in the three main domains, we aimed to create relatively homogeneous subgroups of PD patients with similar functional deficit patterns, instead of conducting univariate regression analyses, where the effects of each main functional variable may be mixed with the confounding effects of other functional domains. Therefore, we performed an unsupervised classification of subjects based on the combination of Stroop interference, JLO, and UPDRS-III scores, which divided PD patients into three subgroups. Figure 2 displays histograms of the three subgroups in relation to the scores of each of the three test scores.

When the resulting three subgroups were scrutinized regarding their scores in the three functional domains, namely executive (Stroop), motor (UPDRS-III), and visuospatial (JLO) functions, the VS_H_/M_H_ subgroup (*n* = 25) displayed high visuospatial function in addition to higher motor function compared to the other two subgroups. Among the other two subgroups, VS_H_/M_L_ (*n* = 18) showed preserved visuospatial function but exhibited lower motor function, whereas VS_L_/M_L_ (*n* = 12) exhibited both lower visuospatial and motor functions compared to the other two subgroups. Although statistically significant differences in Stroop scores were observed between the subgroups (Supplementary Material Table 3), their distributions showed considerable overlap (Fig. 2a), suggesting that Stroop performance contributed less to the symptomatic clustering compared to visuospatial and motor measures. We also examined whether disease duration was associated with the variables used in the clustering algorithm (UPDRS-III, Stroop, and JLO) using Spearman correlation analysis. This analysis revealed a significant relationship between disease duration and UPDRS-III motor scores (*R* = 0.379, *p* = 0.004), indicating that longer disease duration was associated with greater motor deficits, consistent with the progressive nature of PD. However, no significant correlations were found between disease duration and Stroop (*R* = 0.191, *p* = 0.162) or JLO (*R* = -0.031, *p* = 0.819) scores, suggesting that cognitive decline in PD does not exhibit a strictly linear relationship with disease duration.

**Fig. 3:**
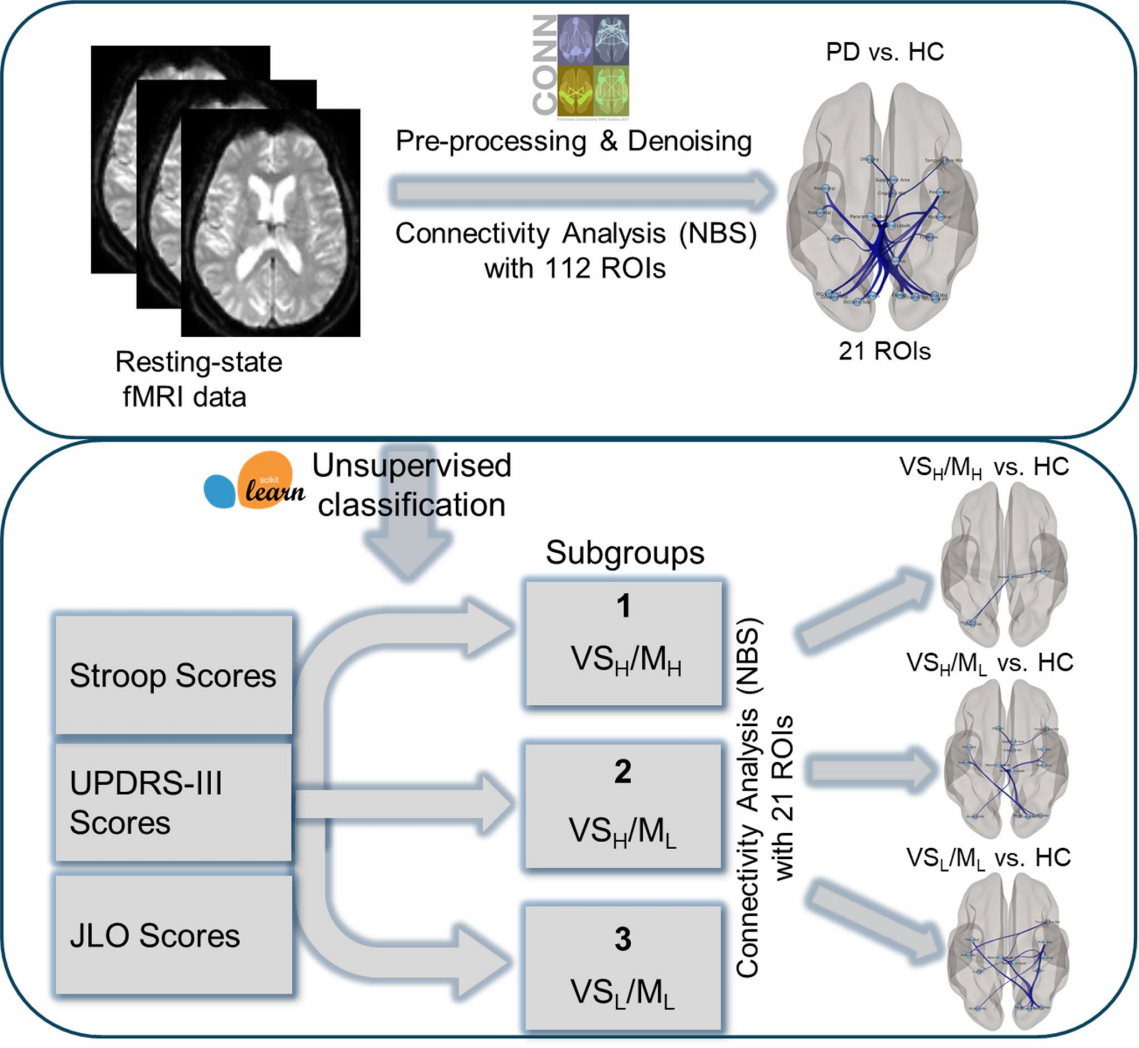
Flowchart of the unsupervised classification and functional connectivity analysis in this study. PD: Parkinson’s Disease; HC: Healthy control; UPDRS-III: Unified Parkinson’s Disease Rating Scale Part III

We compared the FC differences between the HC group and these three subgroups separately, and performed ROI-to-ROI analyses using the NBS method with 21 ROIs significantly affected in the PD-versus-HC group comparison. The unsupervised classification and functional connectivity analysis flowchart of this study is presented in Fig. 3.

**Fig. 4:**
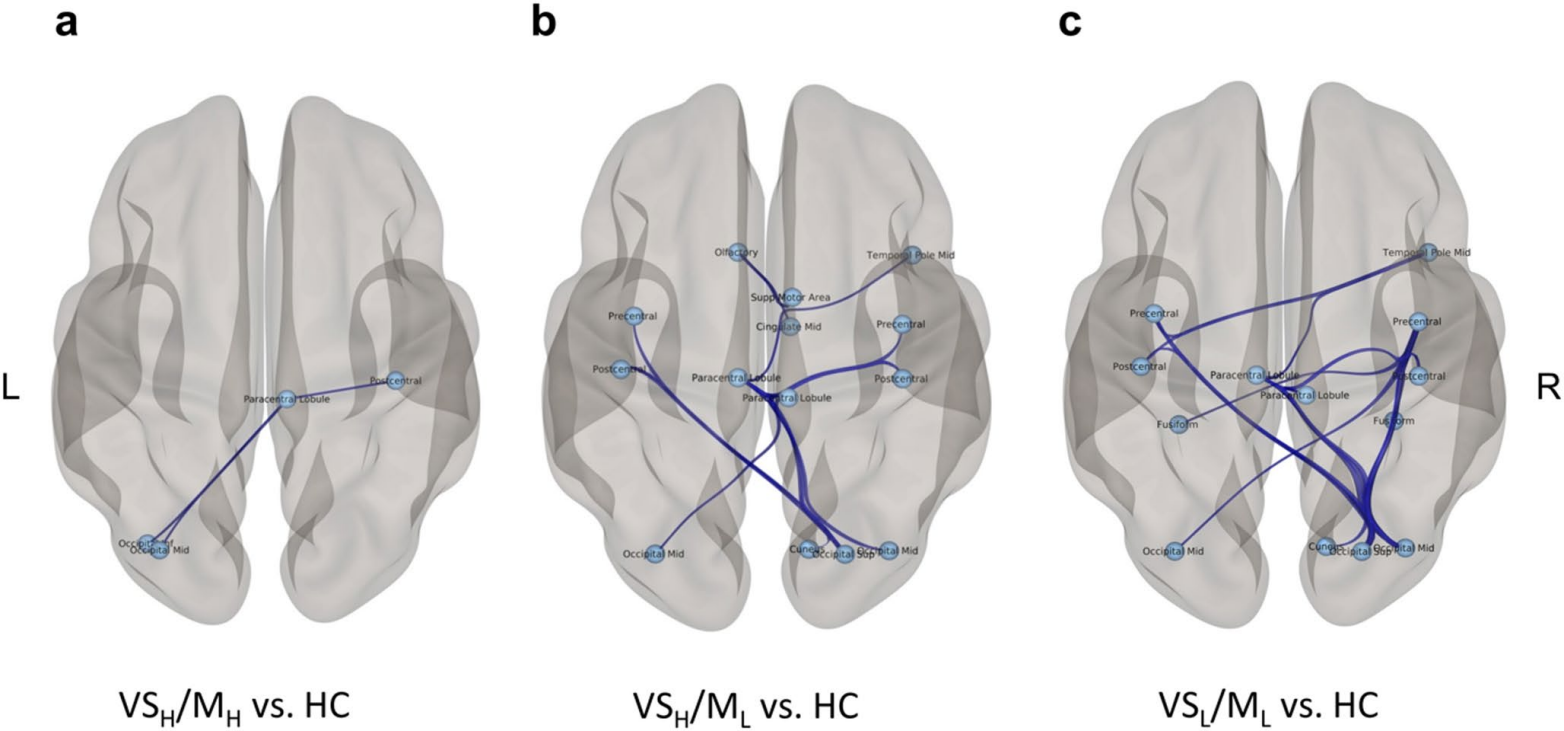
Functional connectivity alterations in the comparison between PD subgroups and HC are shown. **a**) The subnetwork depicts decreased connectivity in the VS_H_/M_H_ subgroup compared to HC. **b**) The subnetwork depicts decreased connectivity in the VS_H_/M_L_ subgroup compared to HC. **c**) The subnetwork depicts decreased connectivity in the VS_L_/M_L_ subgroup compared to HC. Edges between brain regions represent functional connections

Compared to the HC group, the VS_H_/M_H_ subgroup showed significantly decreased FC in six connections among four cortical regions, including the middle and inferior occipital gyri, the paracentral lobule, and the postcentral gyrus (*p* < 0.05, FWE-corrected) (Supplementary Material Table 4, Fig. 4a). Compared to the HC group, the VS_H/_M_L_ subgroup showed significantly decreased FC in 22 connections among 14 cortical regions (*p* < 0.05, FWE-corrected). These connections were within the SMN and between the SMN and VN, including the bilateral middle occipital gyri, the right cuneus, and the right superior occipital gyrus (Supplementary Material Table 5, Fig. 4b). VS_L/_M_L_ subgroup showed significantly decreased FC compared to the HC group in 38 connections among 13 cortical regions, mainly between the SMN and VN areas. These areas included the bilateral middle occipital gyri, the right cuneus, the right superior occipital gyrus, as well as the bilateral fusiform gyri (*p* < 0.05, FWE-corrected) (Supplementary Material Table 6, Fig. 4c).

## Discussion

In this study, we investigated resting-state functional connectivity changes in PD patients by performing a large-scale ROI-to-ROI analysis using 112 cortical and subcortical regions defined by the AAL3 atlas. Utilizing the NBS method, we identified a wide subnetwork showing significantly decreased connectivity in PD patients compared to HCs, which covered extensive visual areas including the superior, medial, and inferior occipital regions, the cuneus, and the fusiform gyrus, as well as sensorimotor regions, including the precentral, postcentral, and paracentral regions, and the SMA, as well as the precuneus, middle cingulate gyrus, left olfactory area, and right middle temporal pole.

Within this PD-associated network, the significantly affected FC pairs were mostly those located within the SMN or those connecting the VN to the SMN. This pattern is generally aligns with motor and visual-motor integration deficits in PD, and has been reported in a number of previous studies [13, 28, 29, 30]. An fMRI study with non-demented PD patients demonstrated reduced FC within and between the SMN and sensory networks, including the VN and auditory network, as well as between the SMN and specific subcortical regions [29]. Another FC study based on ICA showed decreased connectivity within the SMN and between the SMN and VN [30]. This recent report strong aligns with our results, as it reported no significant change in cortico-striatal FC, but found strongly significant cortico-cortical FCs changes, especially between the SMN and VN. While these studies and many other FC studies on PD are consistent regarding the presence and importance of the cortico-cortical FC changes along the disease continuum [7, 10, 31], the specific associations between these connectivity changes and the motor and cognitive features of patient subgroups remain an open question [29, 32].

Attention-executive and visuospatial impairments are frequently reported in PD and often coexist with memory impairments, reflecting the heterogeneous nature of cognitive dysfunction in PD [5]. Because of the heterogeneity of functional deficits in PD, we initially tried to separate the FC patterns of specific symptom domains by performing linear regression analyses between all FC edges and the cognitive and motor scores reflecting motor (UPDRS-III), executive (Stroop), and visuospatial (JLO) functions. In these analyses, we observed a significant regression of executive dysfunction, represented by Stroop scores, with a subnetwork containing the frontal and temporal regions, in addition to the SMN and VN regions. Within this subnetwork, the FC decreases between the inferior and superior medial frontal areas, the orbitofrontal area, and the motor areas were in accordance with the expected disconnection between the executive control network and the SMN. The presence of the SMN and VN regions in this subnetwork, which is regressed to the Stroop scores, points to the fact that the disconnections in the SMN and VN regions may play a role in executive dysfunctions in PD.

On the other hand, no FC pattern specifically regressed to the motor or visuospatial scores. Most likely, the asymmetric distributions and cumulation of the UPDRS-III or JLO scores around specific portions of the scales (Fig. 2) hindered the estimation of a reliable linear regression between motor or visuospatial functional deficits and specific FC subnetworks. Therefore, instead of performing separate regression analyses between each functional domain and FC metrics, we tried to construct data-driven PD subgroups with homogeneous functional characteristics. For this purpose, an unsupervised clustering algorithm was applied to the Stroop, JLO, and UPDRS-III scores of all PD patients. The clustering algorithm revealed three data-driven subgroups, primarily separated based on visuospatial and motor performance, as these dimensions showed clearer between-group distinctions than Stroop performance, which exhibited substantial distributional overlap across the groups. The VS_H_/M_H_ subgroup displayed high visuospatial function in addition to higher motor function compared to the other two subgroups. The VS_H_/M_L_ subgroup was characterized by impaired motor function but preserved visuospatial function, while the VS_L_/M_L_ subgroup exhibited both lower visuospatial and motor function compared to the other subgroups. The FC patterns of these three groups of patients were then compared with that of the HC group. In the VS_H_/M_H_ subgroup, a subnetwork consisting of only six connections between the right paracentral lobule and right postcentral gyrus of the SMN and two VN regions showed reduced connectivity compared to HC. The VS_H_/M_L_ subgroup revealed reduced connectivity in a large subnetwork containing both SMN and VN components, with a larger number of affected connections within the SMN. Finally, the VS_L_/M_L_ subgroup, characterized by lower visuospatial and motor function, showed reduced FC within a large visuomotor network, but this time with more pronounced disconnections between the VN and SMN areas. Specifically, in this subgroup of patients, the connections between the bilateral fusiform gyrus and the precentral gyrus were affected, in contrast to the two other subgroups.

The results of this symptom-based clustering of PD patients can be discussed in the context of the ‘dual syndrome hypothesis’, which proposes that executive and visuospatial dysfunctions represent two cognitive subtypes in PD, and may be important for estimating the risk of progression to PD-D [16–19]. Our results confirm that the symptom patterns of PD patients may allow for the discrimination of specific subgroups. However, the symptomatic clustering in our dataset suggests that the executive deficits are more homogeneously distributed among PD patients, while the severity of the motor and visuospatial dysfunctions serves as the discriminative factors of PD subtypes. The relatively preserved motor and visuospatial functions (VS_H_/M_H_), motor dysfunctions with relatively preserved visuospatial function (VS_H_/M_L_), and the co-existence of both visuospatial and motor dysfunctions (VS_L_/M_L_) appear to be the typical characteristics of the three PD stereotypes. Whether the presence of severe visuospatial deficits serves as a stronger risk factor for PD-D, as discussed in the ‘dual syndrome hypothesis’, is not testable within the scope of the present study, but our results show that patients with both motor and visuospatial deficits (VS_L_/M_L_) display the widest and strongest functional disconnection pattern among all PD patients.

Visual symptoms such as reduced contrast sensitivity, color discrimination, as well as more complex symptoms such as visual hallucinations and impairments in object recognition, visuospatial functions, and motion perception are common among the non-motor symptoms of PD [33]. Some previous fMRI studies showed abnormal FC within the VN and between visual and other cortical and subcortical regions, which might be related to the visual impairments [10, 31]. Our results demonstrate that connectivity changes within the SMN and between VN and SMN regions generally occur in PD, even when the motor or visual symptoms are not in the foreground. This points to a general tendency towards impaired visual-motor integration in the disease, as has been pronounced in other recent studies [29, 30]. Our results extend these previous reports by demonstrating a gradient pattern in which stronger disconnection within the SMN is associated with motor dysfunctions, while stronger VN to SMN disconnections, specifically the fusiform-to-motor connections, are evident in subjects with worse performance in visuospatial functions.

The cortico-striatal connectivity reductions reported in many FC studies on PD were absent in our PD-associated subnetwork [13, 14, 34]. Many previous studies, which focused on the cortico-striatal network in relation to the striatal dopamine depletion in PD, reported significant FC changes between striatal structures and motor cortical areas [12–14, 28]. A study using three striatal seeds and multiple regression analyses by Helmich et al. demonstrated reduced FC between the posterior putamen and the inferior parietal cortex, pointing to decreased sensorimotor integration in PD [13]. Using a graph theory approach, Wu et al. demonstrated decreased FC in the SMA, the left putamen, and the left dorsolateral prefrontal cortex (DLPFC) [12]. Another study by Wu et al. using a seed-to-voxel FC approach reported decreased FC between the SMA and the left putamen [28]. The differences between our results and these previous reports may partly be due to the fact that we recorded medicated PD patients in the ON state, while many previous reports on cortico-striatal disconnections are based on unmedicated patients. It has been argued that cortico-striatal FC changes are reduced or normalized in the ON state [35]. However, from a technical point of view, this is hard to judge because of the presence of excessive movement artifacts in many off-medication patients. On the other hand, the presence of significant cortico-striatal findings in studies carried out in the ON state also suggests that the absence of cortico-striatal findings in the present study cannot be solely attributed to medication effects. We argue that stronger and more consistent cortico-cortical FC changes dominated the NBS results on the whole-brain ROI-to-ROI connectivity, leading to a reduced sensitivity to cortico-striatal FC changes.

Two further interesting FC findings observed in the subnetwork obtained through the group comparison between PD and HC were reduced connectivity between the precuneus and the precentral areas, as well as reduced connectivity between the olfactory area and the supplementary motor (SMA) along with the middle cingulate areas. The precuneus, among its various functions arising from its position within large-scale networks such as the DMN, DAN or FPN, has been shown to play an important role in visuospatial imagery, especially regarding the control of body movements (motor imagery) [36]. Our observation of reduced connectivity between the precuneus and precentral areas, which has not been reported before, may significantly contribute to the impaired control of movements in PD. On the other hand, olfactory dysfunction, an important non-motor, sensory symptom of PD, has been found to correlate with the severity of motor symptoms, cognitive impairment, and disease progression [37–40]. A two-year follow-up study by He et al. revealed that hyposmic PD patients showed worse motor symptoms compared to normosmic PD patients and suggested that olfactory dysfunction might be a useful predictor of subsequent cognitive impairment and disease progression [40]. In our study, we did not objectively evaluate olfactory function in PD patients and thus could not classify them based on their level of olfactory impairment. However, we found a FC reduction between the olfactory cortex and the SMA as well as middle cingulate gyrus of PD patients compared to the HC group, which has not been previously reported. This interesting finding may be associated with the relationship between olfactory dysfunction and motor and cognitive decline in PD.

The present study involves some limitations. First, in the symptom-based subgrouping of the PD patients we classified 55 PD patients into three subgroups, which led to rather small sample sizes in the subgroups. Further studies with larger subgroups may be necessary to generalize the results and demonstrate more specific FC changes, associated with the motor, executive and visuospatial dysfunctions. Second, to minimize tremor-related movement artifacts, fMRI scans, as well as motor and cognitive assessments, were conducted while patients were taking their usual antiparkinsonian medication. A number of task-based and resting-state fMRI studies have reported that dopaminergic medication normalizes connectivity changes in the motor networks of PD patients [12–42]. While decreased cortico-cortical connectivity between the SMN and sensory networks has been widely observed in the OFF-medication state [29], it was still present between the SMN and VN in the ON-medication state [30]. Additionally, other studies have also reported connectivity alterations in the ON-medication state [10, 31, 43], suggesting that dopaminergic therapy does not fully restore normal functional connectivity. Since we did not perform within-subject ON/OFF comparisons, we could not explicitly determine the impact of medication on functional connectivity. Third, another limitation is the use of a limited number of cognitive tests to assess executive and visuospatial functions. Given the extensive MRI acquisition alongside clinical and cognitive assessments, we prioritized feasibility to minimize participant burden and attrition. While a more comprehensive test battery could have provided a broader evaluation, our approach ensured a balance between methodological soundness and practical applicability. Current findings can be further extended by incorporating ON/OFF medication comparisons to better isolate the effects of dopaminergic treatment and by expanding the neuropsychological test battery to enhance the characterization of cognitive subtypes in PD and their relationship with FC alterations.

## Conclusion

Our study, in line with the recent studies, demonstrates that functional disconnection between the SMN and VN in PD patients is a global feature of the disease [29, 30]. By constructing homogeneous functional subgroups of PD patients through data-driven symptom clustering and applying the NBS method for FC analyses, we extend this result by demonstrating that at least two separate routes of functional disconnection may be responsible for the inhomogeneous symptom distribution in PD. Rather than applying predefined cut-off-based clinical classifications, our approach aimed to explore how variability in motor and cognitive performance relates to functional connectivity patterns in PD. By applying a clustering approach based on widely used and well-validated measures for executive, visuospatial, and motor domains, we generated relatively homogeneous subgroups, which allowed us to identfy distinct FC alterations across varying symptom severities. Specifically, more pronounced FC disruptions within the SMN are associated with a predominant motor symptom profile, whereas greater FC disconnection between the VN and SMN correspond to more pronounced visuospatial deficits.

## Electronic supplementary material

Below is the link to the electronic supplementary material.

Supplementary Material 1

## Data Availability

Raw data used in this study are not publicly available to comply with local regulations and to protect individuals’ privacy in accordance with the Turkish Personal Data Protection Law.
