# Supplementary material for: Investigation of symptom-specific functional connectivity patterns in Parkinson’s disease
Source: Neurol Sci. 2025 Jun 14;46(9):4385–96. doi: 10.1007/s10072-025-08287-4 (PMC12394358; doi:10.1007/s10072-025-08287-4)
Supplement: Supplementary file 1 — Supplementary Material 1 [file 10072_2025_8287_MOESM1_ESM.docx]

**﻿** **Investigation of Symptom-Specific Functional Connectivity Patterns in Parkinson’s Disease**

***Supplementary Table 1.*** *Results of the NBS analysis, PD vs. HC*

| **Analysis Unit** | **Intensity** | ***t* (77)** | ***p*-uncorrected** | ***p*-FWE** |
| --- | --- | --- | --- | --- |
| Size = 78 | 295.78 |  | 0.0009 | 0.0026 |
| Paracentral lobule (R) – Occipital Middle (L) |  | -5.18 | 0.0000 |  |
| Paracentral lobule (R) – Postcentral (R) |  | -4.62 | 0.0000 |  |
| Paracentral lobule (R) – Occipital Middle (R) |  | -4.36 | 0.0000 |  |
| Paracentral lobule (R) – Occipital Inferior (L) |  | -4.24 | 0.0000 |  |
| Paracentral lobule (R) – Occipital Superior (R) |  | -4.12 | 0.0000 |  |
| Paracentral lobule (R) – Fusiform (L) |  | -3.93 | 0.0001 |  |
| Paracentral lobule (R) – Occipital Inferior (R) |  | -3.91 | 0.0001 |  |
| Paracentral lobule (R) – Postcentral (L) |  | -3.87 | 0.0001 |  |
| Paracentral lobule (R) – Cuneus (R) |  | -3.71 | 0.0002 |  |
| Paracentral lobule (R) – Precentral (R) |  | -3.58 | 0.0003 |  |
| Paracentral lobule (R) – Occipital Superior (L) |  | -3.56 | 0.0003 |  |
| Paracentral lobule (R) – Temporal Pole Middle (R) |  | -3.54 | 0.0003 |  |
| Paracentral lobule (R) – Fusiform (R) |  | -3.30 | 0.0007 |  |
| Paracentral lobule (L) – Occipital Superior (R) |  | -4.38 | 0.0000 |  |
| Paracentral lobule (L) – Postcentral (R) |  | -4.34 | 0.0000 |  |
| Paracentral lobule (L) – Temporal Pole Middle (R) |  | -4.24 | 0.0000 |  |
| Paracentral lobule (L) – Precentral (R) |  | -4.16 | 0.0000 |  |
| Paracentral lobule (L) – Cuneus (R) |  | -3.86 | 0.0001 |  |
| Paracentral lobule (L) – Occipital Middle (L) |  | -3.83 | 0.0001 |  |
| Paracentral lobule (L) – Occipital Superior (L) |  | -3.51 | 0.0004 |  |
| Paracentral lobule (L) – Occipital Middle (R) |  | -3.42 | 0.0005 |  |
| Paracentral lobule (L) – Occipital Inferior (L) |  | -3.22 | 0.0009 |  |
| Paracentral lobule (L) – Cuneus (L) |  | -3.20 | 0.0010 |  |
| Precentral (R) – Cuneus (R) |  | -4.27 | 0.0000 |  |
| Precentral (R) – Occipital Middle (L) |  | -3.98 | 0.0001 |  |
| Precentral (R) – Cuneus (L) |  | -3.84 | 0.0001 |  |
| Precentral (R) – Occipital Superior (R) |  | -3.64 | 0.0002 |  |
| Precentral (R) – Occipital Middle (R) |  | -3.32 | 0.0007 |  |
| Precentral (R) – Occipital Inferior (L) |  | -3.32 | 0.0007 |  |
| Occipital Superior (R) – Precentral (L) |  | -3.90 | 0.0001 |  |
| Occipital Superior (R) – Postcentral (L) |  | -3.61 | 0.0003 |  |
| Cuneus (R) – Precentral (L) |  | -3.73 | 0.0002 |  |
| Cuneus (R) – Postcentral (L) |  | -3.56 | 0.0003 |  |
| Precentral (L) – Occipital Superior (R) |  | -3.90 | 0.0001 |  |
| Precentral (L) – Precuneus (R) |  | -3.31 | 0.0007 |  |
| Precentral (L) – Occipital Middle (R) |  | -3.28 | 0.0008 |  |
| Precentral (L) – Cuneus (L) |  | -3.25 | 0.0008 |  |
| Cuneus (L) – Supplementary Motor Area (R) |  | -3.32 | 0.0007 |  |
| Olfactory (L) – Cingulate Middle (R) |  | -4.07 | 0.0001 |  |
| Olfactory (L) – Supplementary Motor Area (R) |  | -3.38 | 0.0006 |  |

***Supplementary Table 2.*** *Results of the NBS analysis, effect of Stroop test*

| **Analysis Unit** | **Intensity** | ***t* (53)** | ***p*-uncorrected** | ***p*-FWE** |
| --- | --- | --- | --- | --- |
| Size = 36 | 133.13 |  | 0.0059 | 0.0207 |
| Postcentral (R) – Frontal Inferior Orbital (L) |  | -5.00 | 0.0000 |  |
| Postcentral (R) – Frontal Inferior Tri (L) |  | -4.72 | 0.0000 |  |
| Postcentral (R) – Frontal Inferior Orbital (R) |  | -3.76 | 0.0002 |  |
| Postcentral (R) – Temporal Middle (L) |  | -3.58 | 0.0004 |  |
| Postcentral (R) – Temporal Pole Superior (L) |  | -3.48 | 0.0005 |  |
| Postcentral (R) – Frontal Superior Medial (L) |  | -3.47 | 0.0005 |  |
| Frontal inferior orbital (L) – Postcentral (L) |  | -3.85 | 0.0002 |  |
| Frontal inferior orbital (L) – Calcarine (R) |  | -3.74 | 0.0002 |  |
| Frontal inferior orbital (L) – Occipital Middle (R) |  | -3.56 | 0.0004 |  |
| Frontal inferior orbital (L) – Calcarine (L) |  | -3.53 | 0.0004 |  |
| Frontal inferior orbital (L) – Cuneus (R) |  | -3.51 | 0.0005 |  |
| Postcentral (L) – Frontal Inferior Orbital (R) |  | -3.89 | 0.0001 |  |
| Postcentral (L) – OFCpost (R) |  | -3.61 | 0.0003 |  |
| Postcentral (L) – Temporal Pole Middle (R) |  | -3.61 | 0.0003 |  |
| Postcentral (L) – Frontal Inferior Tri (L) |  | -3.33 | 0.0008 |  |
| Frontal Inferior Tri (L) – Precentral (R) |  | -3.27 | 0.0010 |  |
| Temporal Middle (L) – Supramarginal (R) |  | -3.40 | 0.0006 |  |
| Temporal Pole Superior (L) – Lingual (L) |  | -3.26 | 0.0010 |  |
| Precentral (R) – Frontal Inferior Tri (L) |  | -3.27 | 0.0010 |  |

|  | **HC (*n*=24)** | **VS_H_/M_H_ (*n*=25)** | **VS_H_/M_L_ (*n*=18)** | **VS_L_/M_L_ (*n*=12)** | **Statistics*, P* value** |
| --- | --- | --- | --- | --- | --- |
|  | **Mean (SD)** | **Mean (SD)** | **Mean (SD)** | **Mean (SD)** |  |
| Gender (F/M) | 9/15 | 10/15 | 5/13 | 1/11 | ꭓ^2^ = 4.32, 0.228^a^ |
| Age (year) | 59.79 (7.12) | 59.68 (8.52) | 62.67 (9.12) | 65.58 (7.15) | *F* = 1.91, 0.135^b^ |
| Education (year) | 10.04 (4.03) | 9.56 (3.88) | 10.83 (3.5) | 7.33 (3.6) | ꭓ^2^ = 6.24, 0.100 |
| MMSE | 29.67 (0.81) | 29.04 (1.27) | 29 (0.9) | 27.92 (1.37) | ꭓ^2^ = 20.14, <0.001^c^ |
| GDS | 4.33 (3.37) | 5.76 (4.61) | 6.72 (3.25) | 5 (3.55) | ꭓ^2^ = 4.41, 0.220^c^ |
| Disease Duration (years) | NA | 5 (3.25) | 6.89 (3.46) | 7.58 (4.29) | ꭓ^2^ = 5.11, 0.078^c^ |
| LEDD | NA | 580.27 (258.93) | 827.29 (369.66) | 913.75 (432.17) | *F* = 4.91, 0.011^b^ |
| UPDRS-III | NA | 19.04 (4.86) | 37.5 (8.98) | 36.17 (11.73) | ꭓ^2^ = 34.04, <0.001^c^ |
| Stroop Interference Score | NA | 48.68 (24.9) | 67.11 (22.82) | 88.83 (44) | ꭓ^2^ = 14.73, 0.001^c^ |
| JLO | NA | 24.4 (2.38) | 25.44 (2.22) | 14.67 (4.18) | ꭓ^2^ = 27.47, <0.001^c^ |

***Supplementary Table 3.*** *Demographic and clinical characteristics of PD subgroups (VS_H_/M_H_, VS_H_/M_L_, VS_L_/M_L_)* *and healthy controls (HC)*

SD: Standard Deviation, GDS: Geriatric Depression Scale, MMSE: Mini-Mental State Examination, UPDRS-III: Unified Parkinson’s Disease Rating Scale Part III, LEDD: Levodopa Equivalent Daily Dose (mg/day), JLO: Benton Judgment of Line Orientation

^a^Pearson’s chi-squared test

^b^One-way ANOVA test; Post-hoc pairwise comparisons (Bonferroni-adjusted) revealed the following significant differences: LEDD: VS_H_/M_H_ vs VS_L_/M_L_ (p = 0.021).

^c^Kruskal-Wallis H test; Post-hoc pairwise comparisons (Mann-Whitney U test, Bonferroni-adjusted) revealed the following significant differences: MMSE: HC vs VS_H_/M_L_ (0.007), HC vs VS_L_/M_L_ (<0.001), VS_H_/M_H_ vs VS_L_/M_L_ (p = 0.009); UPDRS-III: VS_H_/M_H_ vs VS_L_/M_L_ (p < 0.001), VS_H_/M_H_ vs VS_H_/M_L_ (p < 0.001), JLO: VS_H_/M_H_ vs VS_L_/M_L_ (p < 0.001), VS_H_/M_L_ vs VS_L_/M_L_ (p < 0.001), Stroop: VS_H_/M_H_ vs VS_L_/M_L_ (p < 0.001), VS_H_/M_H_ vs VS_H_/M_L_ (p = 0.006).

***Supplementary Table 4.*** *Results of the NBS analysis, VS_H_/M_H_ vs. HC*

| **Analysis Unit** | **Intensity** | ***t* (47)** | ***p*-uncorrected** | ***p*-FWE** |
| --- | --- | --- | --- | --- |
| Size = 6 | 21.69 |  | 0.0113 | 0.0126 |
| Paracentral lobule (R) – Occipital Middle (L) |  | -4.07 | 0.0001 |  |
| Paracentral lobule (R) – Postcentral (R) |  | -3.46 | 0.0006 |  |
| Paracentral lobule (R) – Occipital Inferior (L) |  | -3.31 | 0.0009 |  |

***Supplementary Table 5.*** *Results of the NBS analysis, VS_H_/M_L_ vs. HC*

| **Analysis Unit** | **Intensity** | ***t* (40)** | ***p*-uncorrected** | ***p*-FWE** |
| --- | --- | --- | --- | --- |
| Size = 22 | 81.33 |  | 0.0014 | 0.0015 |
| Paracentral lobule (R) – Occipital Superior (R) |  | -3.89 | 0.0002 |  |
| Paracentral lobule (R) – Occipital Middle (L) |  | -3.88 | 0.0002 |  |
| Paracentral lobule (R) – Occipital Middle (R) |  | -3.87 | 0.0002 |  |
| Paracentral lobule (R) – Postcentral (R) |  | -3.62 | 0.0004 |  |
| Occipital Superior (R) – Postcentral (L) |  | -4.09 | 0.0001 |  |
| Occipital Superior (R) – Paracentral lobule (L) |  | -3.82 | 0.0002 |  |
| Occipital Superior (R) – Precentral (L) |  | -3.38 | 0.0008 |  |
| Paracentral lobule (L) – Occipital Superior (R) |  | -3.82 | 0.0002 |  |
| Paracentral lobule (L) – Postcentral (R) |  | -3.73 | 0.0003 |  |
| Paracentral lobule (L) – Temporal Pole Middle (R) |  | -3.43 | 0.0007 |  |
| Paracentral lobule (L) – Precentral (R) |  | -3.31 | 0.0010 |  |
| Postcentral (L) – Cuneus (R) |  | -3.64 | 0.0004 |  |
| Olfactory (L) – Supplementary Motor Area (R) |  | -4.03 | 0.0001 |  |
| Olfactory (L) – Cingulate Middle (R) |  | -3.81 | 0.0002 |  |

***Supplementary Table 6.*** *Results of the NBS analysis, VS_L_/M_L_ vs. HC*

| **Analysis Unit** | **Intensity** | ***t* (34)** | ***p*-uncorrected** | ***p*-FWE** |
| --- | --- | --- | --- | --- |
| Size = 38 | 141.83 |  | 0.0003 | 0.0002 |
| Precentral (R) – Paracentral (L) |  | -4.68 | 0.0000 |  |
| Precentral (R) – Cuneus (R) |  | -4.11 | 0.0001 |  |
| Precentral (R) – Paracentral Lobule (R) |  | -3.95 | 0.0002 |  |
| Precentral (R) – Occipital Superior (R) |  | -3.88 | 0.0002 |  |
| Precentral (R) – Fusiform (R) |  | -3.63 | 0.0005 |  |
| Precentral (R) – Fusiform (L) |  | -3.44 | 0.0008 |  |
| Precentral (R) – Occipital Middle (R) |  | -3.44 | 0.0008 |  |
| Precentral (R) – Occipital Middle (L) |  | -3.43 | 0.0008 |  |
| Occipital Superior (R) – Precentral (L) |  | -4.15 | 0.0001 |  |
| Occipital Superior (R) – Paracentral Lobule (L) |  | -3.50 | 0.0007 |  |
| Occipital Superior (R) – Postcentral (L) |  | -3.43 | 0.0008 |  |
| Occipital Middle (R) – Precentral (L) |  | -4.10 | 0.0001 |  |
| Occipital Middle (R) – Paracentral Lobule (R) |  | -3.44 | 0.0008 |  |
| Occipital Middle (R) – Postcentral (R) |  | -3.43 | 0.0008 |  |
| Occipital Middle (R) – Paracentral Lobule (L) |  | -3.40 | 0.0009 |  |
| Paracentral Lobule (L) – Temporal Pole Middle (R) |  | -4.33 | 0.0001 |  |
| Precentral (L) – Temporal Pole Middle (R) |  | -3.59 | 0.0005 |  |
| Temporal Pole Middle (R) – Postcentral (L) |  | -3.55 | 0.0006 |  |
